# Evaluation of Laboratory Techniques for the Diagnosis of Leptospira‐Associated Equine Recurrent Uveitis (ERU) With Focus on the Goldmann‐Witmer Coefficient

**DOI:** 10.1111/vop.70132

**Published:** 2026-01-10

**Authors:** Lena Kirmse, Katharina Thieme, Marcus Georg Doherr, Johanna Corinna Eule

**Affiliations:** ^1^ Ophthalmology Unit, Centre for Veterinary Clinical Services Freie Universität Berlin Berlin Germany; ^2^ Tierärztliche Praxis für Pferde Stefanie Süß Deinste Lower Saxony Germany; ^3^ Equine Clinic Seeburg Dallgow‐Döberitz Germany; ^4^ Institute of Veterinary Epidemiology and Biostatistics, School of Veterinary Medicine Freie Universität Berlin Berlin Germany

## Abstract

**Purpose:**

To evaluate different laboratory procedures for determining the etiologic diagnosis of equine recurrent uveitis regarding intraocular infection with *Leptospira* spp. and to establish a diagnostic guideline.

**Material and Methods:**

Eighty horses with a history of ERU were ophthalmologically examined. Serum and aqueous humor were collected. Total protein, albumin level, and MAT against *Leptospira* spp. were evaluated on serum and aqueous humor. PCR for *Leptospira* spp., EHV‐1 and ‐4 was performed on aqueous humor. Goldmann‐Witmer coefficient (GWC) and C‐value (CC) were calculated based on MAT. In 42 cases, an additional ELISA was initiated.

**Results:**

Forty‐six female and 34 male horses of different breeds (mean age 10.9 years; range 3 to 31) were included. By MAT 56/80 horses (70.0%) were identified seropositive for *Leptospira* spp. MAT results were positive for *Leptospira* spp. in aqueous humor of 47/80 (58.8%) patients. PCR tested 16/80 (20.0%) positive, ELISA detected 13/42 (31.0%) positive. Neither EHV‐1 nor EHV‐4 were detected by PCR. Calculating GWC gives evidence suggestive of intraocular involvement with *Leptospira* spp. in 53/80 (66.3%) at the level ≥ 3. Setting GWC ≥ 3 as gold standard, ELISA and C ≥ 2 closely matched this threshold, showing high accuracy (95.2%; 91.3%), sensitivity (86.7%; 84.9%), and strong agreement (*V* = 0.90; *V* = 0.81). PCR was less accurate (53.8%) and sensitive (30.2%) compared to GWC.

**Conclusion:**

Within this setting, GWC achieved the highest number of positive results for detecting intraocular involvement of *Leptospira* spp. when compared to PCR, ELISA, and C‐value.

## 1 Introduction

ERU is a worldwide occurring disease and the leading cause of blindness in horses. This immune‐mediated disease is characterized by recurrent episodes of uveitis, leading to progressive intraocular damage and therefore vision loss [1, 2].

The exact etiology is not fully understood yet. Besides an immune‐mediated pathogenesis [2, 3, 4, 5, 6] and genetic predisposition [7, 8, 9, 10, 11, 12, 13, 14], intraocular infection with *Leptospira* spp. has been shown to be a risk factor for the development of ERU, especially in certain geographic regions [15, 16, 17, 18, 19, 20, 21, 22, 23, 24, 25, 26, 27, 28, 29, 30, 31, 32, 33, 34].

Currently, there are no clinical guidelines for the diagnosis of intraocular leptospiral infection in patients with ERU and clinical testing remains highly variable among practitioners.

In humans, who can develop a similar Leptospira‐associated recurrent uveitis, leptospiral DNA has been detected by PCR in aqueous humor [35]. In horses, aqueous and/or vitreous humor is used as material for the detection of leptospiral DNA in chronic ERU [31]. While paracentesis of aqueous humor is less prone to complications [2], PCR on vitreous humor seems to be more sufficient for the detection of leptospiral DNA [20, 31, 36].

In humans, horses, dogs and cattle, antibodies against Leptospira appear within the bloodstream 5–7 days after infection [37, 38, 39, 40, 41] and are detectable in humans beyond acute inflammation [42]. In aqueous humor, antibodies have been detected 2 weeks after experimental infection of rabbits with *Leptospira* spp. [43]. For the diagnosis of Leptospira‐associated ERU, different diagnostic methods and/or ocular fluids have been used in the past with different results depending on cut‐off values that were used: microagglutination test (MAT) titer combined with calculation of the Goldmann‐Witmer coefficient (GWC) [44, 45] and/or C‐value (C) [10, 22, 25, 27, 32] as well as enzyme‐linked immunosorbent assay (ELISA) [34, 46].

The Goldmann‐Witmer coefficient (GWC) is a diagnostic parameter used to evaluate the production of intraocular antibodies against specific infectious agents. Its calculation includes the antibody titers in aqueous humor and serum, as well as the corresponding total protein or immunoglobulin concentrations in both fluids [22, 44, 47, 48, 49, 50]. By accounting for these protein levels, the GWC corrects for variations in the blood‐ocular barrier permeability, providing a more reliable measure of local (intraocular) antibody synthesis than a simple aqueous‐to‐serum antibody ratio [44]. This makes the GWC a valuable tool for identifying infectious etiologies of uveitis [47, 48, 49].

(1) $$C=\frac{\text{antibody titer in serum}}{\text{antibody titer in aqueous humor}}$$

Formula 1: Calculation of aqueous‐to‐serum antibody ratio (referred to as C‐value) [10, 12, 22, 27, 32, 51].

(2) $$\mathrm{GWC}=\frac{\text{antibody titer in serum}}{\text{antibody titer in aqueous humor}}:\frac{\text{total protein in aqueous humor}}{\text{total protein in serum}}$$

Formula 2: Calculation of the Goldmann‐Witmer coefficient (GWC) [44, 52].

The aim of the study was to evaluate the different laboratory procedures for determining the etiologic diagnosis of equine recurrent uveitis regarding intraocular infection with *Leptospira* *spp.* and to establish a diagnostic guideline. Besides that, data on the etiology and signalment of horses presenting to a referral clinic in North‐Eastern Germany are presented.

## 2 Material and Methods

Analysis of medical records of equine patients with recurrent uveitis admitted to the ophthalmology unit of the Equine Clinic, Freie Universität Berlin, Germany between 2015 and 2021.

**FIGURE 1:**
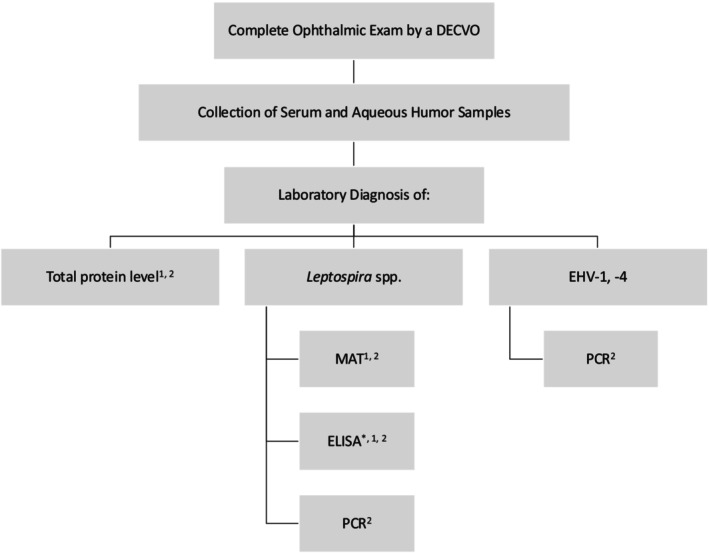
Flow chart of conducted diagnostic work‐up for the etiology of recurrent equine uveitis [1]. Serum was used [2], aqueous humor was used, *only in cases with C < 4.

Complete data on the signalment was extracted from the medical records regarding the following inclusion criteria. Recurrent uveitis was diagnosed based on the history (more than one episode of primary uveitis that was not related to trauma or other ocular diseases) and clinical/ophthalmic findings in patients showing symptoms of acute or chronic uveitis [1, 53, 54, 55, 56, 57, 58, 59]. A complete diagnostic work‐up was performed (see Table 1; Figure 1), including blood sampling (serum), paracentesis of aqueous humor, biochemistry for detection of total protein and albumin in serum and aqueous humor, PCR to detect *Leptospira* spp. and EHV‐1 and ‐4 (aqueous humor), and microagglutination test against different serovars of *Leptospira* spp. (serum and aqueous humor). In cases where a C < 4 was detected, an ELISA against different serovars of *Leptospira* spp. was initiated additionally.

**TABLE 1:** Required laboratory approach for horses with equine recurrent uveitis to be included in the study.

Sample	Examination method	Subject
Serum	Biochemistry	Total protein
		Albumin
	MAT	*Leptospira* spp.
	ELISA^a^	*Leptospira* spp.
Aqueous humor	Biochemistry	Total protein
		Albumin
	PCR	*Leptospira* spp.
		EHV‐1
		EHV‐4
	MAT	*Leptospira* spp.
	ELISA^a^	*Leptospira* spp.

^a^ Only in cases with *C* < 4.

Horses from the breeding associations Holsteiner, Hanoverian, Oldenburg, Westphalian, Trakehner and German Sport Horse were categorized as ‘German Warmblood’ [60, 61, 62, 63]. Horses of the breeds Appaloosa, Knabstrupper, Noriker, Pony of the Americas, and miniature horses were classified under the broader category of horses with potential leopard complex spotting, referred to as ‘leopard‐spotted’ horses [11, 51, 64, 65, 66, 67, 68]. Additional breeds identified separately included Icelandic Horses, American Quarter Horses, and Standardbreds. All other breeds were assigned to the category ‘others’.

Coat colors were not documented and therefore not analyzed.

Vaccination status was available for all animals. None of the presented horses was vaccinated against Leptospira.

### 2.1 Ophthalmic Exam

All reviewed patients underwent ocular examination performed by a DECVO. That included slit‐lamp biomicroscopy (KOWA SL‐17, Kowa Company Ltd.), direct (HEINE BETA 200, HEINE Optotechnik GmbH & Co. KG; PanOptic, Welch Allyn Inc.) and/or indirect (HEINE OMEGA 600, HEINE Optotechnik, GmbH; Welch Allyn Inc.) fundoscopy with a 20D lens (Volk Optical, Tech. Optics Inc.), fluorescein stain, rebound tonometry (Icare TONOVET), and/or ocular ultrasound (Toshiba Aplio 500, 10 MHz linear probe, Toshiba Medical Systems Corporation).

### 2.2 Sample Collection and Processing

Blood was collected from the external jugular vein using a 20G needle and a 12–24 mL syringe prior to intravenous sedation and the whole blood sample was immediately transferred to an empty blood collection tube. For serum separation, the tube was centrifuged within 30 to 60 min after collection as standard procedure.

After instillation of an auriculopalpebral nerve block and ocular surface anesthesia, aseptic preparation of the ocular surface was done [52]. Between 0.5 and 1.5 mL of aqueous humor were collected using a 27G needle and 2 mL syringe via limbal paracentesis [69]. Afterwards, an equivalent amount of preservative‐free lidocaine (Lidocard B. Braun 2%, B. Braun AG) and adrenaline (Adrenalin 1:1000, Jenapharm) were injected into the anterior chamber. Aqueous humor samples were divided into three parts.

### 2.3 Sample Analysis

In serum and aqueous humor samples, total protein and albumin levels were determined in the laboratory of the Small Animal Clinic, Freie Universität Berlin, Germany (Random‐Access‐Analyzer Konelab 60i; Thermo Fisher Scientific).

For the detection of antibody titers, a microagglutination test (MAT) against *L. interrogans* Serovar Canicola, Hardjo, Grippotyphosa, Icterohaemorrhagiae, Bratislava, Pomona, and Pyrogenes was performed on serum and aqueous humor samples at Bayrisches Landesamt für Gesundheit und Lebensmittelsicherheit (LGL) in Oberschleissheim, Germany [45, 46]. A titer ≥ 1:100 was considered as evidential for a previous contact with *Leptospira* spp. [70].

To evaluate the results regarding intraocular antibody production, both the C‐value (see Formula 1) and the Goldmann‐Witmer coefficient (GWC, see Formulas 2 and 4) were calculated.

For patients with antibody titer ≥ 1:50 in aqueous humor but negative antibody titer (1:0) in serum, a scoring code (see Data S1) was defined to overcome the mathematical problem that the aqueous antibody titer cannot be divided by 0 (see Formula 3). C‐value levels ≥ 2 and ≥ 4 were used as thresholds for further statistical analysis [10, 12, 22, 27, 32, 66].

A GWC ≥ 3 was evaluated as positive evidence for intraocular leptospiral infection [44, 71, 72].

For patients with positive antibody titer in aqueous humor, but negative antibody titer in serum, the same scoring code was used (see above and Data S1).
(3)

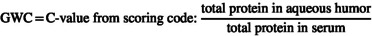




Formula 3: Calculation of the Goldmann‐Witmer coefficient (GWC) based on the C‐value in patients with negative serum microagglutination test (MAT) titers and positive aqueous humor‐MAT.

$${\mathrm{TP}}_{\mathrm{ah}}=7.4\;g/L;{\mathrm{TP}}_{\mathrm{se}}=63.3\;g/L$$

$${\mathrm{MAT}}_{\mathrm{ah}}=1:100;{\mathrm{MAT}}_{\mathrm{se}}=\text{negative}\;\left(1:0\right)$$

C‐value = 4 (see Data S1).

(4) $$\mathrm{GWC}=4:\frac{7.4\;g/L}{63.3\;g/L}=34.22$$

Formula 4: Example for a calculation of the Goldmann‐Witmer coefficient (GWC) based on the C‐value in patients with negative serum microagglutination test (MAT_se_) titers and positive aqueous humor‐MAT (MAT_ah_).

Additionally, an ELISA was performed on serum and aqueous humor samples to determine antibody titers including IgG, IgM and IgA when the C‐value_MAT_ was ≤ 2 and in three patients with C > 2. Routine testing always included the serovar Grippotyphosa. When MAT identified antibodies against other serovars, these were additionally tested. Results were documented by the laboratory in gradations: 0 = negative, 1 = borderline, 2 = weakly positive, 3 = positive, 4 = strongly positive. A positive ELISA result was present when grade 2 (weakly positive) was determined for at least one immunoglobulin class [43]. In this study, the ELISA result was considered positive for intraocular leptospiral infection if the value measured in the aqueous humor was at least two levels higher than the value measured in the serum.

A real‐time PCR for *Leptospira* spp. and equine herpesviruses 1 (EHV‐1) and 4 (EHV‐4) was performed on aqueous humor samples initiated at LABOKLIN (Bad Kissingen, Germany; accredited according to DIN EN ISO 17025:2018).

### 2.4 Data Analysis

Data was analyzed using IBM SPSS 29, with the alpha threshold of 0.05 set for statistical significance. Nominal and ordinal data were compared using chi‐square test and Fisher's exact test. Cramer's V was calculated as the effect size, ranging from 0 (no association) to 1 (perfect association). Similarities between the different thresholds for the interpretation of intraocular antibody production were analyzed using receiver operating characteristics (ROC), as were the sensitivity and specificity of different test methods.

## 3 Results

Eighty horses met the inclusion criteria with a history and clinical signs of recurrent uveitis plus a complete panel of laboratory work‐up as defined in M & M.

### 3.1 Study Population

Patients aged 3 to 31 years (median 10 years). There were 57.5% (46/80) male and 42.5% (34/80) female horses. Regarding breeds, horses were categorized as German Warmblood (*n* = 34; 42.5%), Icelandic Horse (*n* = 10; 12.5%), Standardbred (*n* = 8; 10.0%), American Quarter Horse (AQH; *n* = 3; 3.8%), ‘leopard‐spotted’ (*n* = 5; 8.4%), and ‘others’ (*n* = 21; 26.3%).

### 3.2 Detection of Intraocular Infection

#### 3.2.1 Antibody Detection With MAT

In serum, positive antibody titers against *Leptospira* spp. (≥ 1:100) were identified in 56/80 cases (70.0%). The most frequently detected serovar was Grippotyphosa (37/56; 66.1%), followed by Pomona (15/56; 26.8%) and Icterohaemorrhagiae (13/56; 23.2%). Grippotyphosa was detected as a single serovar in 25 cases. Combinations of two to four different serovars were identified in 21 of the 56 cases (37.5%). Additionally, a titer of ≤ 1:50 was found in 13/80 patients (16.3%). Table 2 shows the number of detected MAT titers against each tested *Leptospira* serovar based on serum samples.

**TABLE 2:** Number of detected titer levels in MAT against *Leptospira* spp. from serum samples (double entries possible).

		*Leptospira* spp. serovars							
		Canicola	Hardjo	Grippotyphosa	Icterohaemorrhagiae	Pomona	Bratislava	Javanica	Pyrogenes
MAT titer	0	62	79	31	53	63	59	71	60
	1:25	0	0	0	0	0	0	0	2
	1:50	10	1	12	14	2	13	3	10
	1:100	2	0	13	10	3	5	3	7
	1:200	3	0	13	2	1	2	2	1
	1:400	0	0	7	1	3	0	1	0
	1:800	1	0	2	0	5	1	0	0
	1:1600	0	0	2	0	3	0	0	0
	1:3200	2	0	0	0	0	0	0	0
	1:6400	0	0	0	0	0	0	0	0

In aqueous humor, an antibody titer of ≥ 1:100 was detected in 47/80 cases (58.8%). Among these, 22/47 patients (46.8%) showed antibodies exclusively against serovar Grippotyphosa, followed by one case each (2.1%) with antibodies against serovars Pomona and Javanica only. Antibodies against multiple serovars were detected in 23/47 cases (48.9%). Additionally, a titer of ≤ 1:50 was found in 7/80 patients (8.7%). The distribution of detected MAT titer levels against all tested *Leptospira* serovars, as determined from aqueous humor sample analysis, is shown in Table 3.

**TABLE 3:** Number of detected titer levels in MAT against *Leptospira* spp. from aqueous humor samples (double entries possible).

		*Leptospira* spp. serovars							
		Canicola	Hardjo	Grippotyphosa	Icterohaemorrhagiae	Pomona	Bratislava	Javanica	Pyrogenes
MAT titer	0	76	78	30	69	58	68	71	75
	1:50	3	1	6	3	5	3	4	1
	1:100	1	1	8	1	3	2	3	1
	1:200	0	0	6	3	2	4	0	1
	1:400	0	0	4	2	0	1	1	0
	1:800	0	0	8	1	3	1	1	1
	1:1600	0	0	7	0	2	0	0	0
	1:3200	0	0	10	1	7	0	0	1
	1:6400	0	0	1	0	0	0	0	0

#### 3.2.2 Antibody Detection With ELISA

ELISA was performed in 42/80 patients (see M&M). Testing included the serovars Grippotyphosa (*n* = 42), Bratislava (*n* = 41), Icterohaemorrhagiae (*n* = 1), and Pomona (*n* = 1) with 41 patients tested for two and one patient tested for three serovars.

In serum, positive results were received in 40/42 cases (95.2%). Antibody titers against the serovars Grippotyphosa and Bratislava were detected in 34/42 cases (85.0%), against the serovars Grippotyphosa, Bratislava, and Pomona in one case (2.5%), and against Grippotyphosa and Icterohaemorrhagiae in one case (2.5%). Antibodies against Bratislava only were found in three horses (7.5%); antibodies against Grippotyphosa only were found in one case (2.5%). For serovar Grippotyphosa, IgM was detected in 36/37 cases (97.3%), IgG in 21/37 cases (56.8%), and IgA in 21/37 cases (56.8%). IgM alone was detected in 8/37 cases (21.6%). For serovar Bratislava, IgM was the most frequently detected antibody (*n* = 35/38; 92.1%), followed by IgA (*n* = 30/38; 78.9%) and IgG (*n* = 19/38; 50.0%).

In aqueous humor, antibodies were detected by ELISA in 13/42 cases (31.0%).

IgA was detected in all cases tested positive for serovar Grippotyphosa (13/13), while IgG was detected in 4/13 samples and IgM in 1/13 samples.

For serovar Bratislava, 8/13 cases were tested positive. In 7/8 patients, IgA was detected exclusively. IgG and IgA were detected together against serovar Bratislava in one case.

In aqueous humor, no positive results were found in the horses tested for serovar Pomona (*n* = 1) and serovar Icterohaemorrhagiae (*n* = 1).

#### 3.2.3 DNA Detection

Leptospiral DNA was detected in aqueous humor via PCR in 16/80 horses (20.0%).

DNA of EHV was detected in the aqueous humor by PCR for EHV‐1 and for EHV‐4 in 0/80 horses each.

#### 3.2.4 Calculation of the C‐Value

The C‐value calculation (see Figure 3) resulted in C ≥ 2 in 45/80 cases (56.3%) and C ≥ 4 in 39/80 cases (48.7%).

For C ≥ 2, serovar Grippotyphosa was the most frequently detected serovar (*n* = 28), followed by serovars Pomona (*n* = 3), Bratislava (*n* = 2), Icterohaemorrhagiae (*n* = 1), Javanica (*n* = 1), and Pyrogenes (*n* = 1).

In 21 cases, C ≥ 2 was detected for a single serovar (Grippotyphosa, *n* = 18; Bratislava, *n* = 1; Javanica, *n* = 1). In 24 cases, C > 2 was detected in various combinations of two to four serovars.

#### 3.2.5 Calculation of the Goldmann‐Witmer Coefficient

Using a Goldmann‐Witmer coefficient calculation (GWC) with a threshold of ≥ 3 (see Formulas 2 and 3), intraocular antibody production against *Leptospira* spp. was identified in 53/80 horses (66.3%).

Serovar Grippotyphosa was the most frequently detected serovar (*n* = 48), followed by serovars Pomona (*n* = 22), Bratislava (*n* = 12), Icterohaemorrhagiae (*n* = 10), Javanica (*n* = 8), Pyrogenes (*n* = 4), Canicola (*n* = 4), and Hardjo (*n* = 2).

In 24/53 cases (45.3%), antibodies were detected exclusively against serovar Grippotyphosa. Serovars Pomona, Javanica, and Bratislava were detected as single occurrences for a GWC ≥ 3 in one case each (1/53; 1.9%). In 13 cases (24.5%), a GWC ≥ 3 was detected for 2 serovars; in 4 cases (7.5%) for 3 serovars; in 4 cases (7.5%) for 4 serovars; in 3 cases (5.7%) for 5 serovars; and in 2 cases (3.8%) for 7 serovars.

#### 3.2.6 Agreement of GWC and PCR

In all cases with positive pathogen detection by PCR in the aqueous humor (*n* = 16), a Goldmann‐Witmer coefficient of at least 3 was detected (16/16, 100.0%). There was a strong agreement between the two (*p* = 0.001; *V* = 0.36).

### 3.3 Agreement of GWC and ELISA

Of 42 horses that received additional ELISA, a GWC ≥ 3 was detected in 15 horses (36.7%). A positive ELISA outcome was confirmed in 13 of those 15 horses (86.7%) with 88.7% agreement in positive detections between GWC and ELISA (*p* < 0.001; *V* = 0.9).

In two cases with a GWC ≥ 3 (13.3%), the ELISA failed antibody detection.

All horses with a GWC < 3 also had a negative ELISA (27/42; 64.3%).

#### 3.3.1 Correlation Between GWC and MAT (Serum)

Serum antibody titers of ≥ 1:100 were detected in 42 of 53 ERU patients with intraocular antibody production confirmed by a GWC ≥ 3 (79.2%). In 11 of 53 samples (20.8%) with a positive GWC, no serum antibodies were detected. For 14 of 27 patients (51.9%) with a negative GWC, serum antibody titers of ≥ 1:100 were identified. For a serum antibody titer of 1:100, ROC analysis indicated a sensitivity of 79.2% and a specificity of 50.0% for diagnosing intraocular infection with *Leptospira* spp., compared to the GWC results. The area under the curve (AUC) was 0.701. There was little agreement between serum antibody titer and GWC ≥ 3 (*p* = 0.001; *V* = 0.28).

#### 3.3.2 Correlation Between GWC and MAT (Aqueous Humor)

Between aqueous humor antibody detection and GWC, a significant agreement with strong association (*p* < 0.001; *V* = 0.85) was observed in 74/80 cases (92.5%). Among these, 47/80 patients (58.8%) tested positive by both the GWC ≥ 3 and antibody titer in the aqueous humor. In 6/80 samples (7.5%) with a positive GWC ≥ 3, the antibody titer in the aqueous humor was below the threshold titer (1:100) at 1:50. None of the patients with a GWC < 3 showed an antibody titer ≥ 1:100 in the aqueous humor.

ROC analysis for a titer ≥ 1:100 in AH demonstrated a sensitivity of 88.7% and a specificity of 100%, with an AUC of 0.91. Strong agreement was detected between aqueous humor antibody titer and GWC ≥ 3 (*V* = 0.85; *p* < 0.001).

#### 3.3.3 Correlation Between GWC and C‐Value

A strong association was detected between GWC and C‐value (C*C* ≥ 2, *p* < 0.001, *V* = 0.81; C ≥ 4, *p* < 0.001, *V* = 0.70).

In 45/53 cases (84.9%) with a positive GWC ≥ 3, a C‐value ≥ 2 was determined. Eight cases had a (false) negative C‐value ≥ 2 despite a GWC ≥ 3 (8/53, 15.1%). No false positive results have been detected. ROC analysis yielded a sensitivity of 84.9% and a specificity of 100% for C ≥ 2, with an AUC of 0.961.

In 39/53 cases (73.6%) with a positive GWC ≥ 3, a C‐value ≥ 4 was determined. Fourteen cases had a (false) negative C‐value ≥ 4 despite a GWC ≥ 3 (14/53, 26.4%).

Sensitivity and specificity for C ≥ 4 were 73.6% and 100%, respectively, with an AUC of 0.961.

A GWC < 3 and C < 2 have been observed together in 27/80 cases (100%). None of these cases were tested positive by PCR or ELISA.

## 4 Discussion

The retrospective analysis of serum and aqueous humor samples showed an agreement between ERU and intraocular infection with *Leptospira* spp. in 66.3% of the cases. This correlation was confirmed for warmbloods and Icelandic horses with ERU, whereas in ‘leopard‐spotted’ horses, *Leptospira* spp. was only detected in connection with ERU in one case.

### 4.1 Seroprevalence

In this study, a seroprevalence of 70% was observed among horses with equine recurrent uveitis (ERU). These results correlate closely with findings from the UK (65.5%) [30] but differ significantly from prior data from Southern Germany (52.2% [73]; 44% [18]), and the US (41.7% [22]; 11% [16]). Differences in titer thresholds across studies, which vary from 1:100 [16, 22, 30] to 1:400 [18, 74], may result in discrepancies and complicate direct comparisons. In Central Europe, general seroprevalence for equines ranges from 1.5% [75] to 58.5% [76], with up to 72.2% in Northern Ireland [77], whereas data from Central Germany [18] using a 1:400 threshold remain difficult to compare to this study's findings. Geographic conditions also impact prevalence, with higher rates in moist (sub‐, tropical, moist) than in arid regions. Differences are particularly striking in countries with seasonal and regional variation like Brazil [78, 79, 80]. Variation in seroprevalence might also be affected by different management practices of horses. Stable feeding can have a protective effect, while open feeding, communal grazing, and access to natural water sources can increase it [74, 81, 82, 83, 84].

### 4.2 Diagnostic Procedure of Intraocular Infection With *Leptospira* spp.

In the present study, the calculation of the GWC yielded the highest detection rate of 66.3% and the lowest proportion of false positive and false negative results between the test methods, compared with the equivalent MAT result from aqueous humor (58.8%), C‐value ≥ 2 (56.3%) and C ≥ 4 (48.8%), and PCR (20.0%).

In this study, intraocular infection with *Leptospira* spp. was detected in 53 out of 80 ERU‐affected horses through GWC calculation and PCR testing. This combined approach is recognized as reliable for the diagnosis of infectious uveitis in human medicine [47, 85] and is recommended for ERU to improve diagnostic accuracy [22, 25]. Based on these results, 66.3% of the studied population likely has a Leptospira‐associated etiology for ERU. The literature reports varied infection rates from 4.2% to 85% [17, 18, 22, 25, 28, 31, 46], which may stem from differences in detection methods and diagnostic interpretation, such as the antibody titer levels used as diagnostic cutoffs.

The PCR test for detecting *Leptospira* spp. has long been considered a faster and more sensitive alternative to time‐consuming culturing methods [1, 17, 19, 20, 86]. In studies on human infectious uveitis [85, 87, 88, 89] and equine intraocular leptospirosis [17, 18, 19, 25, 31, 46, 90], no positive DNA results were found in healthy control groups. Therefore, some authors suggest that any positive detection of leptospiral DNA in equine ocular samples should be considered a possible intraocular infection [91], but it should be noted that PCR does not determine the pathogen's viability [92].

In the current study, 20.0% of patients showed positive leptospiral DNA in aqueous humor samples via PCR. In the past, detection rates have varied depending on the sample medium used, with aqueous humor samples yielding positive results in 41% to 70% of cases [17, 27, 45, 46, 93], while vitreous samples reported detection rates from 45% to 100% [19, 20, 24, 26, 36]. Differences in detection rates are attributed to varying inclusion criteria for analysis. Previous studies have shown that PCR results from vitreous samples taken during or directly after acute uveitis episodes are often positive in the detection of *Leptospira* spp. Those differences between both fluids might be explained by the continuous production and flow rate of aqueous humor compared to vitreous humor [31] as well as shifts in the distribution ratio between the media [46]. While an even distribution of leptospires in the eye is assumed at the onset of an intraocular infection [43], they seem to hide into the vitreous body as the disease progresses [20, 72]. This may be related to an affinity of leptospires for the gelatinous structure of the vitreous body [31] and their encapsulation in biofilms, which may mask them and contribute to their persistence in the vitreous body [33, 90], potentially leading to false‐negative PCR results.

Although vitreous body samples are proposed as superior for detecting intraocular leptospires when compared to aqueous humor samples [33], false negatives cannot be completely ruled out due to the non‐homogeneous distribution of leptospires in the gelatinous vitreous. In the present study, a preoperative vitreous puncture was not performed due to the existing risks, which is why aqueous humor obtained by paracentesis under standing sedation was used for preoperative diagnostics [17, 22, 25, 31]. In the past, several studies have used vitreous for diagnostic purposes during therapeutic vitrectomy [19, 20, 26, 28, 34]. Horses with intraocular evidence of *Leptospira* spp. benefit from vitrectomy in up to 81% of cases [94, 95], whereas horses with ERU without leptospiral involvement tend to relapse after vitrectomy in 86% of cases [88]. ERU patients with laboratory results being available only after completion of vitrectomy are, under these circumstances, confronted with vitrectomy under general anesthesia and the associated risks with no prospect of success [2]. The use of SNAP Lepto (IDEXX Laboratories Inc.) is recommended as a preoperative test to detect antibodies to leptospiral major outer membrane protein LipL32 [36]. Both aqueous and vitreous humor can be used. The authors argue that in case of a positive test result, sampling and vitrectomy can be performed under general anesthesia. In the case of a negative result, further diagnostics would be unavoidable, and the animal would be released from anesthesia without direct surgery [36]. Additionally, variations in PCR results can arise not only from different sample materials but also from PCR methods. Various real‐time qPCRs have been established to detect different genes associated with leptospirosis, with the LipL32 gene frequently being analyzed in PCR testing [31, 36, 46, 96]. Significant discrepancies in detection rates have been noted with different assays; some studies reported over 70% positive results [17, 20], while others failed to detect leptospiral DNA [22], possibly due to diverse local serovars and inadequate differentiation between pathogenic and saprophytic leptospires [97].

Although the combination of PCR and GWC is recommended for infectious uveitis [25, 47, 48, 49, 85], in the present study PCR did not add further diagnostic value beyond GWC ≥ 3. We acknowledge, however, that a positive PCR result in combination with an elevated GWC may increase specificity and can therefore strengthen the evidence for causative leptospiral involvement in ERU. The apparent discrepancies between both methods should also be interpreted in light of the natural history of infectious diseases. In human leptospirosis, it is well known that the 7‐to‐14‐day phase of acute bacteremia is followed by a phase of increasing antibody production [98]. IgM is produced first, followed by IgG [42, 99]. In human serum, antibody titers could be determined from day 5 to 7 post infection [97, 100], while in horses experimentally infected with serovar Pomona, serum antibodies could be detected from day 9 post infection [101]. The onset of intraocular antibodies differs between pathogen and affected species. In human toxoplasmosis, intraocular antibodies appeared 2 weeks after experimental inoculation [102, 103], whereas no such data are available for human ocular leptospirosis. In rabbits, intraocular antibodies were detected 2 weeks after experimental infection with *Leptospira* spp. [43], whereas in horses no antibodies against *Leptospira* spp. serovar Kennewicki could have been detected within 60 days after experimental infection [39]. As antibody titers increase, the number of leptospires in the blood decreases [104]. If this course of infection is transferred to uveitis, and if it is also considered that leptospires get surrounded by a biofilm by time [90, 95], PCR testing during an early infection period seems to be appropriate [42, 72, 105]. Negative PCR results in the presence of positive intraocular antibody titers could theoretically also be explained by the absence of antigen.

In addition, patients in this study were mostly presented after multiple episodes of uveitis, and aqueous paracentesis was likely performed in quiescent eyes rather than in eyes with acute inflammation, which may be another reason for the low detection rates in PCR.

Due to its high sensitivity and specificity for certain serovars and/or serogroups, the microagglutination test (MAT) titer is established as the gold standard for serodiagnosis of human leptospirosis uveitis [106]. Since antibodies can persist for several years after a leptospiral infection, a more than 4‐fold increase in titer and/or seroconversion is considered evidence of acute leptospirosis [37, 107]. Regarding a Leptospira‐associated pathogenesis of recurrent uveitis in horses, the question arises whether intraocular antibody production can be distinguished from the transfer of antibodies between serum and aqueous humor due to an impaired blood‐ocular barrier. For this reason, the Goldmann‐Witmer coefficient, a ratio of antibody titer in serum to antibody titer in aqueous humor with consideration of the total protein content of both fluids [41], was established.

In the past, the pure ratio of MAT in aqueous humor and serum, also known as C‐value, was repeatedly calculated [10, 12, 22, 32]. Unfortunately, this calculation anchors the risk of misinterpretation, as protein levels are neglected, and the serum titer and the intraocular titer might be equally high in the case of intraocular antibody production. This is confirmed in the current study where the C‐value yielded significantly lower detection rates of intraocular antibody production when compared to GWC ≥ 3 as the chosen reference method. Depending on the chosen cut‐off value (C ≥ 4 and C ≥ 2), a considerable proportion of cases would still have been classified as false negatives. Therefore, it might be concluded that choosing a high C‐value threshold does not increase the sensibility but increases the risk of false negative detection of intraocular antibody production, as it still neglects intraocular protein levels that can be 12.8‐fold higher in diseased in comparison to healthy animals.

While MAT does not allow any statement to be made about the immunoglobulin type present, this can be reliably determined by ELISA. In addition to PCR and MAT, an ELISA was initiated in 42/80 cases. Both IgG and IgM were increasingly detected in intraocular samples from horses suffering from ERU, while neither IgG nor IgM was detected in the eyes of healthy horses [108, 109, 110, 111]. Besides that, IgA is reliably detectable in horses with ERU [112, 113, 114] and is produced intraocularly rather than IgG or IgM [108].

In the present study, the ELISA was considered positive if the result of the aqueous humor sample was two levels higher than that of the serum sample (IgG, IgM and/or IgA C_ELISA_‐Value ≥ 4). A positive result was detected in 13/42 cases. IgA was detected in 13/13 horses with a positive ELISA C‐value ≥ 4. This result is in line with the recommendation to use IgA for the detection of intraocular leptospiral infections due to its high sensitivity [43, 103, 108]. Additionally, all 13 horses with positive IgA detection also had a GWC ≥ 3.

A complete agreement between PCR, MAT, GWC ≥ 3 and ELISA testing was found in only 2/13 cases. In both cases, a positive IgA content for the serovars Grippotyphosa and Bratislava was determined by ELISA. This might be explained by an overlap between bacteremia and immune phase. However, this seems unlikely as Leptospira‐associated uveitis in horses is not a concomitant symptom of acute leptospirosis and only occurs months to years after infection [115, 116]. During human leptospirosis, IgA could be detected in the serum in up to 100% of patients during the first year, whereas both IgM and IgG were detectable for a much shorter time [93]. In horses with ERU, a negative correlation between a positive PCR result in the aqueous humor and the detection of immunoglobulins was found [43]. If the theory of leptospiral masking by biofilm in the vitreous is pursued, the occasional release of leptospires from the biofilm and their passage back into aqueous humor appears to be reasonable for a positive PCR result with simultaneous occurrence of IgA.

**FIGURE 2:**
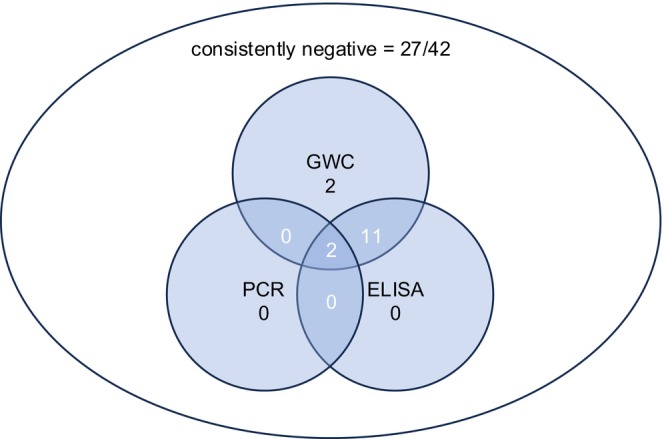
Venn diagram correlating GWC ≥ 3 with aqueous humor PCR and aqueous humor ELISA in 42 horses with ERU, tested by PCR, MAT, and ELISA.

In the present study, 27/29 patients with a negative ELISA (IgG, IgM and/or IgA C_ELISA_‐value < 4) also had a negative PCR result and a GWC_MAT_ < 3. In 2/29 patients, ELISA failed to detect intraocular antibody detection while the GWC_MAT_ was ≥ 3 (see Figure 2).

A GWC ≥ 3 in all samples tested positive by ELISA confirms the GWC as a reliable method for the detection of intraocular antibodies. Although the results of the ELISA do not provide any additional information compared to the calculation of the GWC, it is useful in determining the Ig isotype. In the future, it would be interesting to investigate the feasibility of an ELISA‐based GWC (GWC_ELISA_).

Due to a missing gold standard, concerns about false positive detections via GWC calculation exist, particularly when serum antibodies passively diffuse into the eye in the context of a disrupted blood–ocular barrier, without true local antibody production. Therefore, the GWC should always be interpreted in the clinical context as follows: a combined approach of GWC and, ideally, supported by complementary diagnostic methods such as PCR or culture to reduce the risk of misclassification.

### 4.3 Coincidence Between EHV‐1 and ‐4 Infection and Uveitis

Although none of the tested samples revealed the presence of equine herpes viruses 1 and 4, there has been a long‐standing rumor about their involvement in chorioretinitis in horses and llamas [117, 118, 119, 120, 121]. Therefore, testing for EHV‐1 and ‐4 might be indicated in doubtful cases.

### 4.4 Guideline for the Diagnosis of Leptospira‐Associated ERU

**FIGURE 3:**
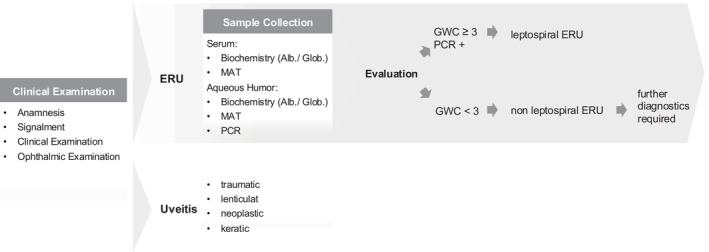
Guideline for the work‐up in all horses with recurrent, chronic uveitis. If a clear association to trauma, keratitis, lenticular changes and/or neoplasia can be made, a thorough work‐up may be neglected. If additional methods, for example, for genetic analysis, detection of ELA subtypes etc., become available in the future, regular use of these methods is endorsed.

Based on the results of this study, a diagnostic guideline was developed to differentiate Leptospira‐associated ERU from recurrent uveitis of other etiologies (see Figure 3). In addition to a complete history and clinical examination for the diagnosis of ERU according to the ACVO/ECVO guidelines, this includes laboratory diagnostics to determine the GWC_MAT_ [2, 61] and PCR. Serum collection and paracentesis should be performed under standing sedation or general anesthesia. The GWC_MAT_ is calculated based on the biochemical analysis of total protein or total globulin content of serum and intraocular fluid, and a determination of antibody titers (MAT) against *Leptospira* spp. from both samples. A GWC_MAT_ ≥ 3 is considered evidential for intraocular antibody production against *Leptospira* spp. and thus a Leptospira‐associated etiology of ERU. Combination with PCR increases diagnostic specificity and helps to avoid misclassification. With GWC_MAT_ < 3, further diagnostics (e.g., ELISA, observation of the present immunoglobulin isotypes) should be initiated, depending on the signalment of the affected patient. If further methods, for example, for genetic analysis, detection of ELA subtypes, etc., were established and become available on the market in the future, it would make sense to use these methods regularly for all horses with ERU, especially with regard to heredity and breeding hygiene.

## 5 Limitations

The study design presents some limitations that should be acknowledged. The retrospective nature of the study introduces potential biases due to the reliance on previously collected data. A limitation of our study is the relatively small sample size (80 patients), which may restrict the generalizability of the findings and reduce statistical power compared to larger cohorts. Nevertheless, each case underwent complete diagnostic processing and thorough data analysis, ensuring high data quality and internal validity. This comprehensive approach strengthens the reliability of the observed associations despite the limited cohort size.

The study was conducted at a referral and second opinion center (University clinic). Owners that present their animals in such facilities might be more receptive to comprehensive diagnostic approaches than others who don't. This creates a selection bias.

The diagnostic workup of the patients presented followed distinct in‐house rules, as described within the Material and Methods section. As a result, there was a strong pre‐selection with respect to ELISA testing, which could introduce bias. However, based on the results presented, this bias could be neglected.

## 6 Conclusion

In regions with a high seroprevalence of *Leptospira* spp., a possible intraocular infection needs to be ruled out regarding the pathogenesis of ERU.

According to the results of the present study, the calculation of the GWC_MAT_ seems to be sufficient to detect intraocular antibody production and is therefore recommended to differentiate between leptospiral and non‐leptospiral ERU. Further studies on causal relationships between the diseases' different etiologies are required.

## Author Contributions

**Johanna Corinna Eule:** data curation, supervision, conceptualization, writing – review and editing, methodology. **Lena Kirmse:** writing – original draft, methodology, formal analysis, resources, conceptualization, investigation, visualization, software. **Katharina Thieme:** writing and review editing, data curation, methodology. **Marcus Georg Doherr:** writing and review editing, methodology, software, formal analysis.

## Funding

Lena Kirmse was supported by the Dahlem Research School (Freie Universität Berlin) and the Elsa‐Neumann‐Stipendium (scholarship).

## Disclosure

The authors have not used AI to generate any part of the manuscript.

## Ethics Statement

This study complies with the Guidelines for Ethical Research in Veterinary Ophthalmology (GERVO) and is exempt from approval by an ethics committee.

## Conflicts of Interest

The authors declare no conflicts of interest.

## Supporting information


**Data S1:** Supporting Information.

## Data Availability

The data that support the findings of this study are available from the corresponding author upon reasonable request.
