# Supplementary material for: Evaluation of Laboratory Techniques for the Diagnosis of Leptospira‐Associated Equine Recurrent Uveitis (ERU) With Focus on the Goldmann‐Witmer Coefficient
Source: Vet Ophthalmol. 2026 Jan 10;29(1):e70132. doi: 10.1111/vop.70132 (PMC12790138; doi:10.1111/vop.70132)
Supplement: Supplementary file 1 — Data S1: Supporting Information. [file VOP-29-0-s001.docx]

Supplementary Material 1: Evaluation code for calculation of GWC when serum titer (MAT_se_) was negative and aqueous humor titer (MAT_ah_) against *Leptospira* serovar was positiv.

| reciprocal MAT_se_ | reciprocal MAT_ah_ | C-value | difference MAT_se_/MAT_ah_ |
| --- | --- | --- | --- |
| 0  50 | 50  50 | 2  1 | 1  0 |
| 0  50  100 | 100  100  100 | 4  2  1 | 2  1  0 |
| 0  50  100  200 | 200  200  200  200 | 8  4  2  1 | 3  2  1  0 |
| 0  50  100  200  400 | 400  400  400  400  400 | 16  8  4  2  1 | 4  3  2  1  0 |
| 0  50  100  200  400  800 | 800  800  800  800  800  800 | 32  16  8  4  2  1 | 5  4  3  2  1  0 |
| 0  50  100  200  400  800  1600 | 1600  1600  1600  1600  1600  1600  1600 | 64  32  16  8  4  2  1 | 6  5  4  3  2  1  0 |
| 0  50  100  200  400  800  1600  3200 | 3200  3200  3200  3200  3200  3200  3200  3200 | 128  64  32  16  8  4  2  1 | 7  6  5  4  3  2  1  0 |
| 0  50  100  200  400  800  1600  3200  6400 | 6400  6400  6400  6400  6400  6400  6400  6400  6400 | 256  128  64  32  16  8  4  2  1 | 8  7  6  5  4  3  2  1  0 |
